# Ubiquitin-Specific Protease 49 Interacts with Bax to Modulate Apoptosis

**DOI:** 10.3390/ijms27094102

**Published:** 2026-05-03

**Authors:** Hae-Seul Choi, Soo-Yeon Kim, So-Ra Kim, Kwang-Hyun Baek

**Affiliations:** 1 Department of Bioconvergence, Graduate School, CHA University, Seongnam 13488, Republic of Korea; chlgotmf01@naver.com; 2 Department of Biomedical Science, Graduate School, CHA University, Seongnam 13488, Republic of Korea; ksysk92217@gmail.com (S.-Y.K.); sora89824@gmail.com (S.-R.K.)

**Keywords:** B-cell lymphoma 2 (Bcl-2) family, deubiquitinating enzyme, deubiquitination, ubiquitination

## Abstract

Bax, a key member of the B-cell lymphoma 2 (Bcl-2) protein family, is essential for inducing mitochondrial apoptosis. In this study, we employed yeast two-hybrid screening to identify ubiquitin-specific protease 49 (USP49) as a binding partner of Bax. Subsequent immunoprecipitation and glutathione S-transferase (GST) pull-down assays confirmed their direct interaction. Functional assays showed that USP49 reduces Bax polyubiquitination at multiple lysine residues within ubiquitin, with the strongest effects observed on K11, K29, K33, and K63 linkages. In contrast, its effect on K48-linked ubiquitination was weak and insufficient to influence Bax protein stability, indicating that USP49 does not regulate Bax abundance through proteasomal degradation. Instead, RT-qPCR analysis revealed that USP49 overexpression significantly increased Bax mRNA levels, and this effect was maintained under apoptosis stimuli (UV, H_2_O_2_, and STS), indicating transcriptional regulation largely independent of stress-induced damage, whereas its effect was modest and not statistically significant under starurosporine treatment. Collectively, these findings demonstrate that USP49 regulates Bax primarily through K29/K33/K63-linked ubiquitination and transcriptional upregulation, highlighting its role as a stress-responsive modulator of apoptosis and a potential therapeutic target in cancer. Moreover, under DNA damage condition (UV), USP49 overexpression marked enhanced apoptosis.

## 1. Introduction

The B-cell lymphoma 2 (Bcl-2) protein family has a crucial role in governing intrinsic apoptosis, which is a pivotal cellular process involved in maintaining cellular homeostasis. This family can be classified into two groups: proapoptotic and anti-apoptotic proteins [1]. Anti-apoptotic members function by preventing the release of mitochondrial apoptotic factors, such as cytochrome c, from the mitochondria, whereas proapoptotic members promote apoptosis by inducing mitochondrial outer membrane permeabilization (MOMP) and the subsequent release of apoptotic factors into the cytosol [2]. Abnormal regulation of the Bcl-2 family has been associated with disruptions in cellular homeostasis and the onset of tumorigenesis [1].

Belonging to the proapoptotic group, Bax is a critical member of the Bcl-2 protein family. Under normal conditions, Bax exists as a monomer in the cytosol. However, upon exposure to cellular stress, Bax is activated through interactions with proapoptotic BH3-only proteins [3]. Activated Bax then translocates to the surface of mitochondria and integrates into the mitochondrial outer membrane (MOM) [4]. Subsequent homo-oligomerization of Bax leads to the formation of pores in the MOM [4], facilitating the release of the proapoptotic molecule cytochrome c and triggering apoptosis [5]. Mutations in the gene encoding Bax can reduce susceptibility of cells to undergo apoptosis [5]. Moreover, the expression level of Bax has been linked to malignant transformation, tumor progression, and metastasis, with low Bax expression considered a negative prognostic factor in cancer [6,7]. Notably, aggressive human prostate cancer has been associated with decreased Bax degradation [8]. Additionally, in preclinical models of acute myeloid leukemia (AML), Bax trigger site activator 1 (BTSA1) has been shown to activate Bax, leading to apoptosis even in cells that are resistant to cell death [9].

Ubiquitination, a process involving the attachment of ubiquitin to substrate proteins through the E1, E2, and E3 enzymes, serves as a crucial regulatory mechanism for numerous intracellular proteins [10]. Following the addition of ubiquitin to a substrate, further ubiquitin molecules can bind to the existing ubiquitin and form polyubiquitin chains at one of seven lysine residues on the ubiquitin protein (K6, K11, K27, K29, K33, K48, or K63), determining the fate of the substrate protein [11,12]. The degradation of substrates via the proteasome primarily occurs through K48-linked polyubiquitin chains. In contrast, K63-linked polyubiquitin chains play roles in regulating receptor endocytosis, translation, DNA damage response, stress response, and lysosomal targeting [12,13]. In contrast, deubiquitinating enzymes (DUBs) can reverse ubiquitination through deubiquitination, effectively removing ubiquitin from target proteins [14]. DUBs, belonging to the ubiquitin system, are critical regulators involved in various biological processes, and are categorized into nine subfamilies: cysteine proteases that contain ubiquitin-specific protease (USP), ubiquitin C-terminal hydrolase (UCH), Machado-Josephin domain (MJD), ovarian tumor protease (OTU), permutated papain fold peptidases of dsDNA viruses and eukaryote (PPPDE), motif interacting with Ub-containing novel DUB family (MINDY), Jab1/MPN/Mov34 metalloenzyme (JAMM), monocyte chemotactic protein-induced proteases (MCPIPs), and ZUFSP/ZUP1 subfamily [15,16,17]. The coordinated actions of ubiquitination and deubiquitination are essential for maintaining protein homeostasis [18].

The pro-apoptotic activity and protein stability of Bax are tightly regulated by the ubiquitin-proteasome system (UPS). Several E3 ubiquitin ligases have been identified as key negative regulators that suppress Bax-mediated apoptosis through distinct mechanisms. For instance, IBR domain-containing protein 2 (IBRDC2 (also known as RNF144B)), an IBR-type E3 ubiquitin ligase, has been characterized in mouse embryonic fibroblasts and neuronal contexts as a regulator of Bax ubiquitination that controls the threshold of apoptosis activation [19]. Parkin has been reported to mediate Bax ubiquitination, thereby sequestering Bax in the cytosol and preventing its translocation to the mitochondria, ultimately inhibiting cytochrome c release [20]. Furthermore, a recent study identified tripartite motif containing 17 (TRIM7) as a critical E3 ligases that promotes K48-linked polyubiquitination of Bax, leading to its proteasomal degradation and subsequent suppression of cell death in gastric cancer cells [21].

In contrast, DUBs can reverse these modifications. While the E3 ligases described above act as negative regulators of Bax, only a limited number of DUBs have been characterized as their functional counterparts. For example, ubiquitin-specific protease 24 (USP24) has been shown to promote apoptosis by deubiquitinating Bax and increasing its stability [8,22]. Additionally, Ku70 has been reported to possess DUB activity towards Bax, and negatively regulating Bax-mediated apoptosis by inhibiting its translocation of Bax from the cytosol to the mitochondria [23]. In contrast, acetylation of Ku70 promotes Bax activation and apoptosis [24], while ubiquitin-specific protease 22 (USP22) downregulates Ku70 acetylation, thereby inhibiting Bax-mediated apoptosis [25]. Our group also recently reported that ubiquitin-specific protease 12 (USP12) regulates the K63-linked ubiquitination of Bax in cervical cancer using HeLa cells [26].

Despite these findings, the regulation of Bax by DUBs remains incompletely understood, particularly regarding lysine-specific ubiquitin linkages beyond the well-studied K48 and K63. To systematically identify novel DUBs that modulate Bax, we previously conducted a comprehensive yeast two-hybrid (Y2H) screening using Bax as bait. From this unbiased approach, we identified several USP family members, including USP12 and a novel uncharacterized candidate, USP49, whose functional relationship with Bax has not been explored [27]. In this present study, we focus on USP49 to elucidate its regulatory role in Bax-mediated apoptosis. To investigate this, we employed complementary cellular models tailored to specific experimental purposes. HEK293T cells were used for biochemical interaction assays, including IP and glutathione S-transferases (GST) pull-down assays, due to their high transfection efficiency and suitability for protein interaction studies. Furthermore, HeLa and HCT116 cells representing cervical and colorectal cancer models, respectively, were employed to validate the functional relevance of USP49 in apoptotic signaling. Our finding reveals that USP49 reduces Bax ubiquitination, with K29, K33, and K63 linkages, but only weak effects on K48, consistent with no change in Bax protein stability. Instead, USP49 markedly increased Bax mRNA expression under basal conditions, and this transcriptional upregulation was sustained across different apoptotic stimuli, indicating that its effect occurs largely independent of stress exposure. Collectively, these findings highlight USP49 as a distinct regulator of Bax, acting through non-degradative ubiquitin linkage and transcriptional upregulation, and suggest its potential as a therapeutic target in apoptosis-related cancer therapy.

## 2. Results

### 2.1. Identification of USP49 as a Binding Partner of Bax

**Figure 1:**
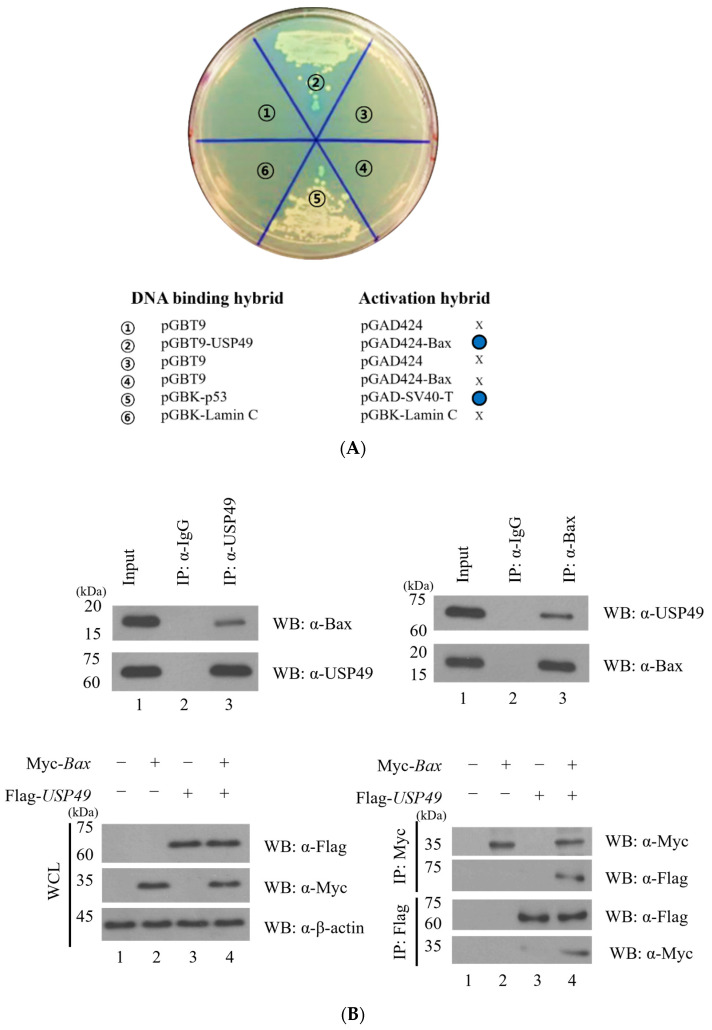

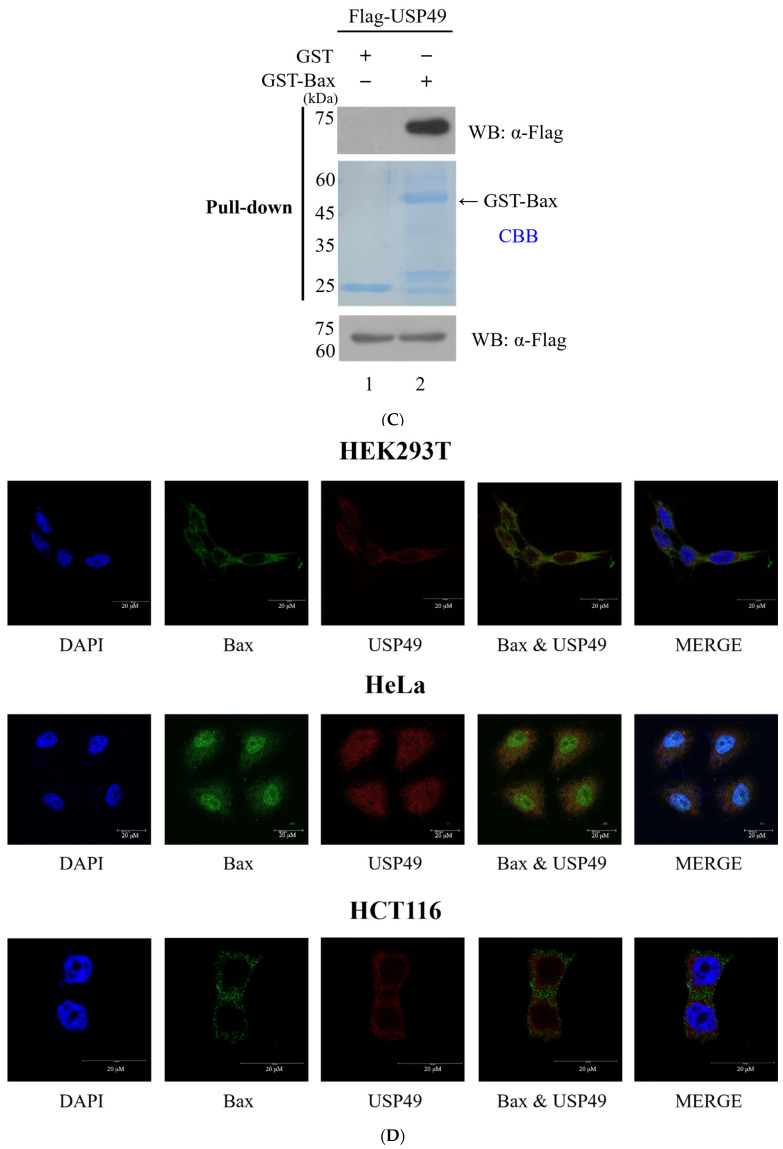
Identification of USP49 as a Bax-binding protein. (**A**) Yeast strain AH109 containing Gal4 DNA-binding domain (BD) plasmids (*USP49*, *Lamin C*, *p53*, and Gal4-BD) was co-transformed with Gal4 activation domain (AD) plasmids (*Bax*, SV40-large T antigen, or Gal4-AD). Colony color was assessed after 3 days of incubation at 30 °C. Gal4-BD and Gal4-AD served as negative controls, while p53 and SV40 large T antigen were used as positive controls. The blue circle indicates binding which was confirmed through screening. (**B**) Cell lysates were immunoprecipitated with an anti-Flag or an anti-Bax antibody, followed by Western blotting with the indicated antibodies. Immunoprecipitation was performed using either an anti-USP49 or an anti-Bax antibodies. Myc-Bax consistently appears at ~33–35 kDa on Western blots; the plasmid was verified by DNA sequencing to ensure the correct sequence. (**C**) Recombinant GST or GST-Bax proteins were incubated with lysates from cells overexpressing Flag-USP49, and bound proteins were analyzed by immunoblotting with an anti-Flag antibody. (**D**) Co-localization of USP49 and Bax was visualized through immunocytochemical staining in HEK239T, HeLa, and HCT116 cell lines. Cells were immunostained with anti-USP49 (red) and anti-Bax (green) antibodies. Nuclei were counterstained with DAPI (blue). Scale bar = 20 μm.

To investigate potential Bax-interacting DUBs, we performed a yeast two-hybrid screening using the DUB enzyme subfamily, specifically focusing on the USP family members. The cDNAs of various USP family members were subcloned into the pGBT9 vector containing the activation domain (AD), while *Bax* was subcloned into the pGAD424 vector containing the binding domain (BD). Upon coexpression of pGBT9-USP49 and pGAD424-*Bax*, yeast transformants exhibited blue colonies, indicative of transcriptional activation of the reporter gene, thus suggesting an interaction between USP49 and Bax (Figure 1A). Coexpression of USP49 and Bax resulted in the development of blue-colored yeast colonies, demonstrating their interaction. As positive controls, yeast cells coexpressing p53 and SV40 large T-antigen also displayed a blue color, indicating successful activation of the reporter gene. In contrast, negative controls, including pGBT9, pGAD424, or pGBT9-Lamin C, did not exhibit a blue color. These results confirm the absence of non-specific reporter gene activation caused by the expression of these vectors. Notably, the pGBT9 vector showed no affinity for Bax, while the pGAD424 vector did not interact with USP49, further substantiating the specific and positive interaction between USP49 and Bax. Overall, our findings establish USP49 as a binding partner of Bax, as evidenced by the positive and specific interaction observed in yeast two-hybrid assays. Further characterization of this interaction will provide valuable insights into the regulatory mechanisms involving Bax and USP49 in apoptotic pathways.

To validate the interaction between USP49 and Bax, we conducted a series of immunoprecipitation assays. The endogenous interaction between USP49 and Bax was demonstrated in HEK293T cells, confirming their physiological binding. Furthermore, co-immunoprecipitation in HEK293T cells overexpressing Flag-USP49 and Myc-Bax further demonstrated that USP49 indeed interacts with Bax, reinforcing initial findings (Figure 1B). Myc-tagged Bax, although predicted to be approximately 26 kDa, consistently migrated at ~33–35 kDa on SDS-PAGE. The integrity of the construct was confirmed by DNA sequencing, and this migration pattern was reproducibly observed across all experiments. This interaction was independently validated through a GST pull-down assay, which confirmed the direct binding between USP49 and Bax (Figure 1C, Supplementary Figure S1). Moreover, immunocytochemical staining in HeLa and HCT116 cells revealed co-localization of USP49 and Bax, providing further support for their functional association (Figure 1D). Collectively, these results strongly indicate the interaction between USP49 and Bax.

### 2.2. Mapping the Interaction Domains Between USP49 and Bax

**Figure 2:**
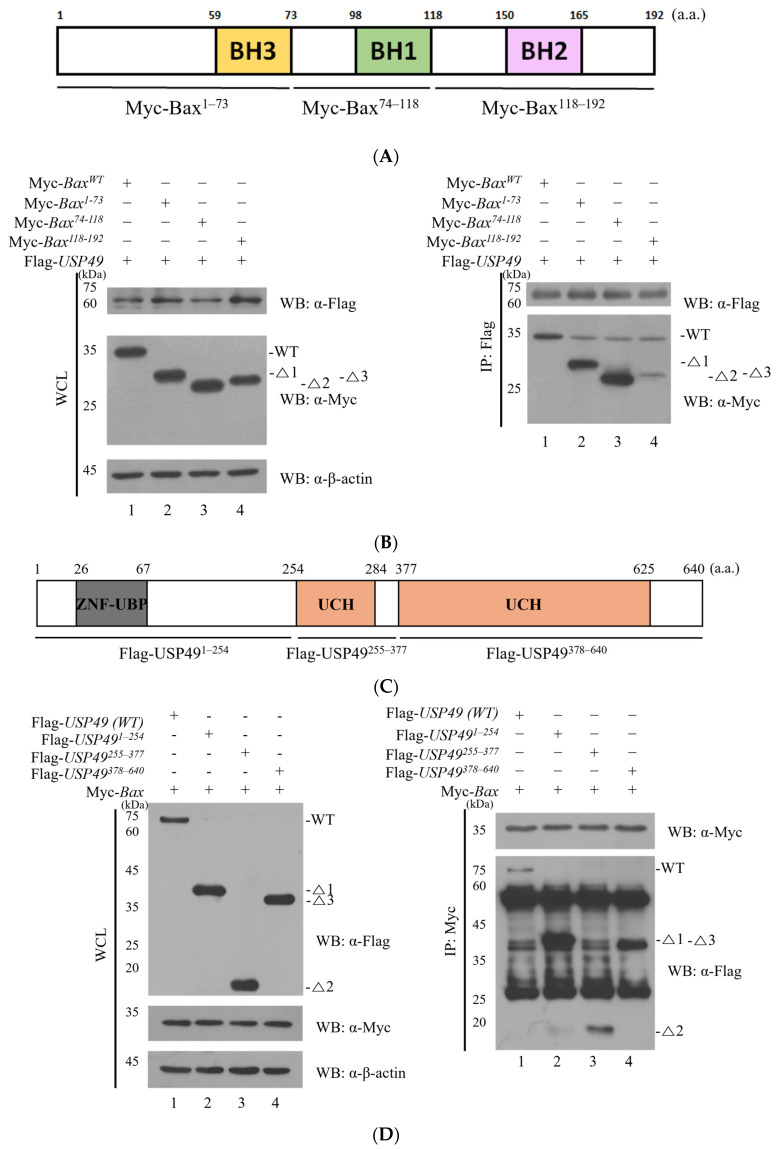
Mapping the binding regions between USP49 and Bax. (**A**) Schematic representation of Bax protein domains (BH1= green, BH2= pink, and BH3 = yellow). (**B**) Flag-*USP49* and various deletion mutants of Myc-*Bax* were transfected into HEK293T cells. Immunoprecipitation was performed with an anti-Flag antibody, and the precipitated proteins were analyzed by Western blotting. (**C**) Schematic representation of USP49 protein domains (ZNF-UBP = gray, UCH = orange). (**D**) Deletion forms of Flag-USP49 were overexpressed in HEK293T cells, followed by immunoprecipitation with an anti-Bax antibody. The precipitated proteins were analyzed by western blotting.

Next, we sought to identify the specific domain of Bax responsible for its interaction with USP49. To achieve this, we generated a series of Bax deletion mutants of Bax and analyzed their binding to USP49 (Figure 2A). As shown in Figure 2B, Flag-USP49 interacted with all Bax deletion forms, suggesting that USP49 binds to multiple regions of Bax or that its primary interaction site is located within the domains shared among these mutants (Figure 2B). Although a faint band at approximately 33 kDa was observed in the mutant lanes, this signal was identified as non-specific background, as a similar band was also detected in the control lane without WT Myc-Bax expression (Supplementary Figure S2). In contrast, the specific signal for WT Myc-Bax was substantially more intense, confirming the specificity of the interaction. These findings suggest that the BH1, BH2, and BH3 domains of Bax are involved in its interaction with USP49. Moreover, to explore the binding capacity of USP49, we generated deletion forms of USP49 and performed binding assays with full-length Bax (Figure 2C). The results revealed that all domains of USP49 were capable of binding to Bax (Figure 2D), further supporting the robust interaction between USP49 and Bax. Taken together, the results from immunoprecipitation, GST pull-down, immunocytochemical staining, and deletion mutant analyses provide compelling evidence for the interaction between USP49 and Bax.

Immunoprecipitation of full-length USP49 using an anti-Flag antibody revealed an interesting finding, as it demonstrated that all deletion forms of Bax were capable of binding to the full-length USP49 (Figure 2B). To further investigate the interaction, we performed a reciprocal immunoprecipitation assay using an anti-Myc antibody. Remarkably, the results showed that all Bax domains interacted with the full-length USP49 (Figure 2B). These findings suggest that the BH1, BH2, and BH3 domains of Bax are involved in its interaction with USP49. Moreover, to explore the binding capacity of USP49, we generated deletion forms of USP49 and performed binding assays with full-length Bax (Figure 2C). The results revealed that all domains of USP49 were capable of binding to Bax (Figure 2D), further supporting the robust interaction between USP49 and Bax. Taken together, the results from immunoprecipitation, GST pull-down, immunocytochemical staining, and deletion mutant analyses provide compelling evidence for the interaction between USP49 and Bax.

### 2.3. USP49 Is a Deubiquitinating Enzyme of Bax

**Figure 3:**
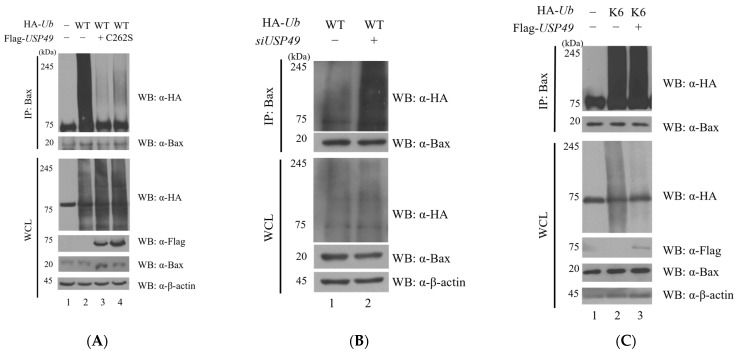

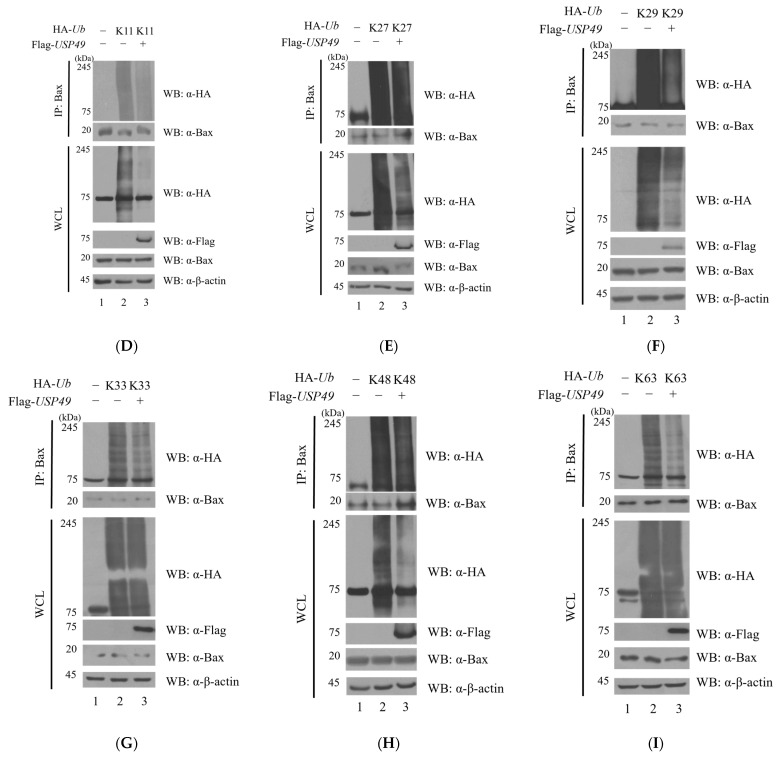
Ubiquitination and deubiquitination of Bax. (**A**) HEK293T cells were transfected with Myc-*Bax* and HA-*Ub* in the presence or absence of Flag-*USP49*. Bax ubiquitination was analyzed by IP and WB under denaturing conditions. (**B**) Effect of USP49 knockdown on Bax ubiquitination. Cells were transfected with control siRNA or *USP49*-targeting siRNA. (**C**–**I**) Linkage-specific deubiquitination of Bax by USP49. HEK293T cells were co-transfected with Flag-*USP49*, Myc-*Bax*, and various HA-tagged *ubiquitin* lysine-only mutants: (**C**) K6, (**D**) K11, (**E**) K27, (**F**) K29, (**G**) K33, (**H**) K48, and (**I**) K63. Ubiquitination levels were determined by IP using anti-Bax antibodies followed by immunoblotting with anti-HA antibodies. Β-actin was used as a loading control for whole cell lysates (WCL).

A previous report has shown that Bax undergoes ubiquitination [10]. To investigate whether USP49 regulates Bax ubiquitination, we performed immunoprecipitation assays with HA-tagged ubiquitin. As shown in Figure 3A, USP49 significantly reduced the polyubiquitination of Bax. To determine whether this effect depends on its catalytic activity, we used a catalytically inactive mutant USP49 (C262S). While wild-type USP49 significantly decreased Bax ubiquitination, the C262S mutant failed to do so, indicating that Bax deubiquitination is dependent on the enzymatic activity of USP49 (Figure 3A). Conversely, siRNA-mediated knockdown of USP49 increased Bax ubiquitination levels (Figure 3B), suggesting that endogenous USP49 is required for maintaining the deubiquitinated state of Bax. This effect was further validated in a dose-dependent manner (Supplementary Figure S3). To identify the ubiquitin linkage types targeted by USP49, we employed ubiquitin mutants allowing only a single lysine linkage (K6, K11, K27, K29, K33, K48, and K63) (Figure 3C–I). USP49 reduced Bax ubiquitination across multiple linkage types, including K11, K29, K33, K48, and K63 (Figure 3D,F–I), indicating broad linkage specificity. Among these, the strongest effects were observed at K29, K33, and K63, whereas the effect on K48 was relatively weak. Since K48-linked chains are primarily associated with proteasomal degradation, this finding is consistent with our observation that Bax protein stability remained largely unchanged in Flag-USP49-overexpressing HEK293T cells (Figure 3) in HEK293T cells. These results indicate that USP49 primarily modulates Bax function through non-degradative linkages rather than regulating its proteasomal degradation.

### 2.4. Effect of USP49 on Bax mRNA and Protein Expression

**Figure 4:**
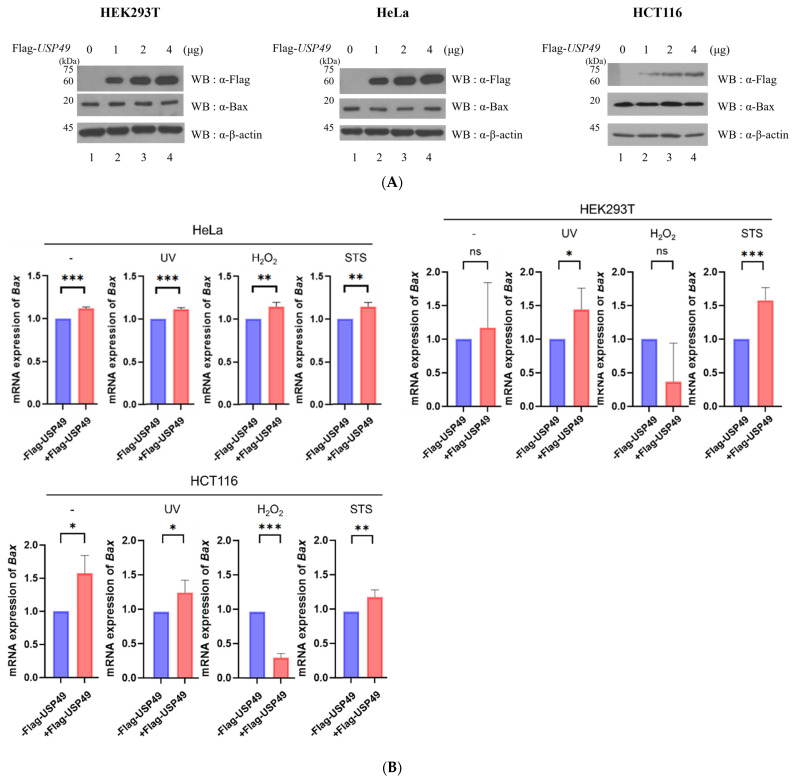
Regulation of Bax by USP49. (**A**) Myc-Bax levels increased in a dose-dependent manner of Flag-USP49 expression in HEK293T, HeLa, and HCT116 cells. (**B**) Bax expression in HeLa, HEK293T, and HCT116 cells overexpressing Flag-USP49 following exposure to UV, H_2_O_2_, or STS (blue = control and red = Flag-*USP49*-transfected samples) (*n* = 3). All quantitative data are presented as mean ± SD. * *p* < 0.05, ** *p* < 0.01, and *** *p* < 0.001 indicate statistically significant differences. Ns: not significant.

To determine whether USP49 regulates Bax expression, we analyzed both protein and mRNA levels. Western blot analysis showed that Bax protein levels remained largely unchanged across multiple cell lines following USP49 overexpression (Figure 4A). However, RT-qPCR analysis revealed that the effect of USP49 on *Bax* mRNA expression is highly dependent on the cell type and the specific apoptotic stimulus. In HeLa cells, USP49 overexpression significantly increased *Bax* mRNA levels under basal and stress conditions, including UV irradiation, H_2_O_2_ treatment, and STS exposure (Figure 4B). In contrast, in HEK293T cells, this effect was primarily observed under UV and STS treatment, whereas in HCT116 cells, *Bax* mRNA levels increased under basal, UV, and STS conditions, but decreased following H_2_O_2_ exposure (Figure 4B). These findings suggest that while USP49 acts as a positive regulator of Bax transcription, its regulatory role is nuanced and modulated by the cellular context and the nature of the apoptotic stimuli.

### 2.5. USP49 Regulates Apoptosis Through Bax Modulation

**Figure 5:**
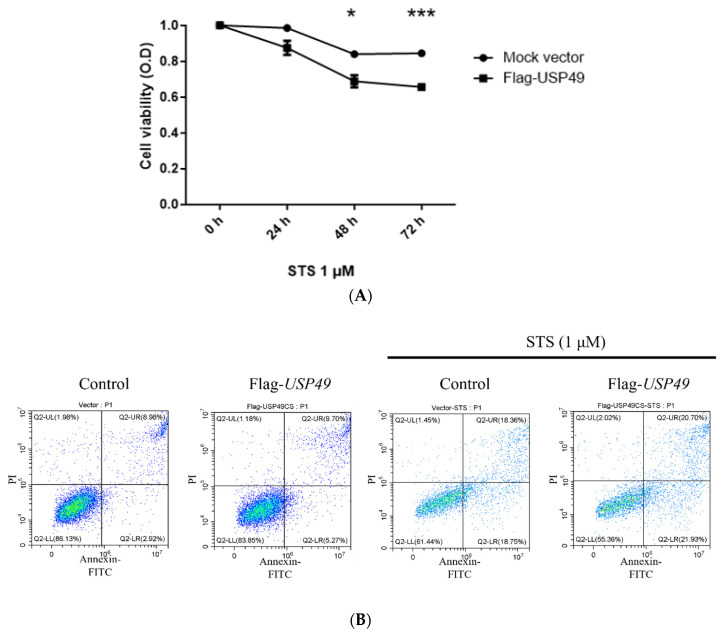

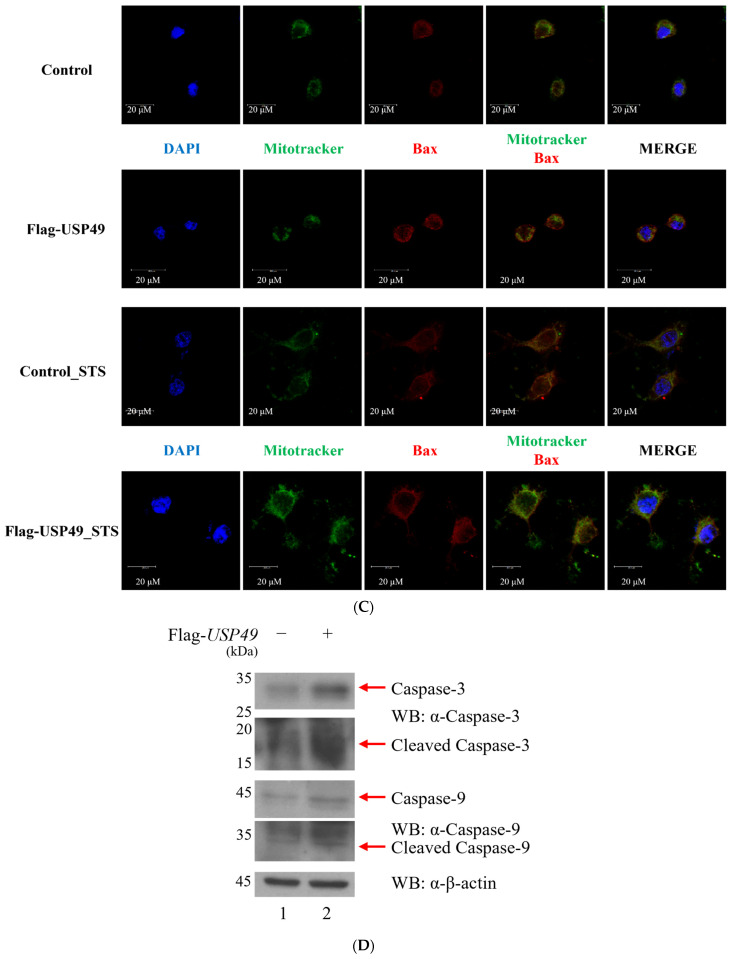
Regulation of Bax by USP49 during apoptosis. (**A**) Cell viability was assessed using a CCK-8 assay in HeLa cells treated with STS, with or without Flag-USP49 overexpression. Cells overexpressing USP49 exhibited reduced viability compared to controls. Data are presented as the mean ± SD of three independent experiments. * *p* < 0.05, *** *p* < 0.001. (**B**) FACS analysis of apoptosis in HeLa cells treated with STS. (**C**) Subcellular localization of Bax. HeLa cells were transfected with Flag-USP49 and treated with 1 μM STS for 4 h to induce early apoptosis. Cells were then stained with anti-Bax antibodies (red) and MitoTracker (green). Nuclei were counterstained with DAPI (blue). Scale bars represent 20 μm. (**D**) Western blot analysis of caspase-3 and caspase-9. HeLa cells were transfected with either an empty vector or Flag-*USP49* for 24 h, and the levels of total and cleaved caspase-3 and caspase-9 were analyzed by Western blotting. β-actin was used as a loading control.

To assess the functional consequences of USP49 expression, we evaluated apoptosis in HeLa cells. CCK-8 assays showed reduced cell viability upon USP49 overexpression under STS treatment (Figure 5A). FACS analysis revealed a modest increase in apoptotic cells in USP49-overexpressing conditions; however, this change did not reach statistical significance (*p* > 0.05) (Figure 5B). Immunofluorescence analysis demonstrated that USP49 promotes Bas translocation to mitochondria. Under STS treatment, USP49-overexpressing cells exhibited pronounce colocalization of Bax with MitoTracker, whereas control cells showed a more diffuse distribution. (Figure 5C). Consistently, Western blot analysis showed increased levels of cleaved caspase-3 and caspase-9 upon USP49 overexpression (Figure 5D), indicating activation of the apoptotic cascade following USP49-mediated Bax translocation. Collectively, these findings suggest that USP49 enhances apoptosis in a stimulus-dependent manner, potentially through transcriptional regulation and subcellular redistribution of Bax rather than changes in its protein stability.

## 3. Discussion

Post-translational modification (PTM) plays a crucial role in the regulation of most proteins, and ubiquitination, a well-known PTM, controls the function of target proteins [28]. Ubiquitination is a cellular process where ubiquitin molecules are attached to substrate proteins [29]. This modification can be reversed by DUBs that detach ubiquitin molecules from substrate proteins [30]. Ubiquitin forms chains through the process of attaching to the lysine residues of another ubiquitin molecule. The type of chain formed can have varying effects on the target protein and can regulate several cellular processes within the UPS. Depending on the type of chain formed, ubiquitination can influence proteasomal degradation, DNA damage response, activation of transcription factors, cell division, and other cellular functions. These chains can be linked in different ways, such as K48-linked chains, which typically target proteins for proteasomal degradation, or K63-linked chains, which often play roles in signaling and DNA damage repair. The regulation of USP has been found to affect various intracellular actions, making it a critical mechanism in cell biology. The Bcl-2 family, which regulates intrinsic apoptosis, is an important pathway targeted in cancer therapy [31]. Disruption of the balance between anti-apoptotic and proapoptotic proteins within the Bcl-2 family can inhibit apoptosis [32]. Abnormal expression of Bax, a proapoptotic member of the Bcl-2 family, has been associated with cancer and influences cancer prognosis [33,34]. In breast cancer cells, low expression of Bax leads to the inhibition of apoptosis [35]. Furthermore, proteasomal degradation of Bax has been linked to a poor prognosis in chronic lymphocytic leukemia [36]. Therefore, modulating the expression of Bax could be a valuable strategy for cancer treatment.

USP49, a member of the USP family, plays a crucial role in histone H2B regulation through deubiquitination and is involved in mRNA splicing [37]. In addition, USP49 is associated with the FKBP51-AKT signaling pathway [38]. By stabilizing FKBP51, USP49 inhibits tumorigenesis and sensitizes pancreatic cancer cells to chemotherapy [38]. A study has revealed that USP49 regulates the stability and transcriptional activity of p53. It was demonstrated that USP49 is induced by DNA damage and that its expression increases in a p53-dependent manner, forming a positive feedback loop between USP49 and p53. USP49 has been reported to regulate p53 activity at both the protein and transcriptional levels through epigenetic control of the MDM2–p53 axis [39]. Moreover, in a mouse model with inhibited USP49, an increase in colorectal cancer incidence was observed, suggesting that USP49 may act as a potential tumor suppressor [40]. However, there has also been a study suggesting that USP49 may play a critical role in mediating drug resistance to 5-Fu and doxorubicin in colorectal cancer cells through the c-MYC-USP49-BAG2 axis [41]. Consistent with this context-dependent behavior, USP49 has also been reported to promote tumor progression in hepatocellular carcinoma by stabilizing RACK1 and enhancing lipid metabolism-driven proliferation and migration, indicating that USP49 may function as an oncogenic regulator in specific cellular contexts [42]. Bax can be phosphorylated at S184 in an AKT-dependent manner [43]. The AKT pathway suppresses the conformational change in Bax and its translocation to the mitochondria [44,45]. USP49 has been shown to play a direct role in DNA double-strand break repair by deubiquitinating γH2AX, thereby modulating 53BP1 recruitment and cellular sensitivity to DNA damage [46]. These include promoting esophagogastric junction adenocarcinoma progression via activation of the SHCBP1-β-catenin-GPX4 axis and inducing gastric cancer malignancy through yes-associated protein 1 (YAP1) stabilization [47,48]. These findings suggest that USP49 may be involved in a broader range of cell survival and death mechanisms beyond the regulation of Bax stability, including the DNA damage response, cell division, the tumor microenvironment, and stress responses. Similar to USP49, USP12 was also identified as a DUB of Bax, but USP12 does not regulate Bax protein levels and has instead been proposed to modulate through deubiquitination [26]. Recent studies indicate that USP12 contributes to immune cell infiltration in the tumor microenvironment (TME) and regulates anti-tumor immune responses through CCL5 expression and its association with Bax [49]. This suggests that USP12 may function as an immune response regulator beyond its role in apoptosis regulation. In this study, USP49 acted as a DUB for Bax but did not appear to regulate Bax stability. This suggests that USP49 may have additional functional targets beyond Bax or that its role in Bax regulation is more complex than previously understood. Further studies are necessary to ascertain the functional significance of USP49 in diverse cellular contexts, including apoptotic pathways, immune responses, and stress responses.

Our findings indicate that USP49 overexpression is associated with an increase in apoptosis, although the effect did not reach statistical significance. This trend was observed under basal conditions and became more evident upon STS treatment, suggesting that USP49 may sensitize cells to apoptotic stimuli without exerting a strong pro-apoptotic effect. Importantly, the DUB activity of USP49 does not affect the expression level of Bax through K48-linked polyubiquitin chains, nor does it regulate Bax degradation through the proteasome, as previously reported [8]. Moreover, USP49 reduced K63-linked polyubiquitination of Bax. USP49-mediated removal of K63-linked ubiquitin chains has been reported in immune signaling contexts, suggesting that USP49-driven K63 deubiquitination broadly functions in non-proteolytic stress and signaling pathways [50]. Although Bax expression levels remained unchanged, apoptosis was still significantly enhanced, indicating that USP49 influences apoptosis signaling through mechanisms independent of Bax stability or expression [51]. To further investigate the specificity of USP49, we analyzed its effect on various Bax polyubiquitin linkages. Our results revealed that USP49 selectively regulated K11-, K29-, K-33-, K48-, and K63- linked polyubiquitin chains of Bax, but does not affect K6- and K27-linked chains. These findings suggest that USP49 may modulate Bax function or apoptotic signaling by regulating K11-, K29-, and K33-linked polyubiquitination, in addition to the well-known roles of K48- and K63-polyubiquitin chains. Among these, the most pronounced effects were observed at K29-, K33-, and K63-linked polyubiquitin chains, which are primarily associated with stress signaling and non-proteasomal degradation rather than protein degradation. K11-linked polyubiquitination has also been implicated in protein turnover as well as transcriptional regulation, cell division, and the cell cycle [52,53]. Collectively, these findings suggest that USP49 modulates Bax function and apoptosis mainly through non-degradative ubiquitin signaling, with its impact becoming evident under specific apoptotic conditions.

In summary, this study demonstrates that USP49 interacts with Bax and selectively regulates its polyubiquitination. Although apoptosis was not significantly increased, a consistent trend was observed, particularly under STS treatment. These results suggest that USP49 may act as a fine-tuner of Bax function rather than a robust pro-apoptotic driver and raise the possibility that it also acts through additional mechanisms or pathways. Future investigations should aim to elucidate the molecular mechanisms of USP49-mediated Bax regulation, validate its role in vivo, and identify other substrates or interacting partners that contribute to its broader cellular functions. The development of USP49-targeting modulators may provide new opportunities for therapeutic intervention in apoptosis-related diseases.

Recent studies have increasingly highlighted the role of DUBs, including USP12 and USP49, not only in regulating protein stability but also in regulating stress responses and immune evasion in cancer cells. This line of research suggests that the USP49-Bax axis may function as a regulator of the tumor microenvironment, as well as regulating stress conditions and apoptosis.

## 4. Materials and Methods

### 4.1. Cell Culture and Transfection

HEK293T and HeLa (cat. no. CRL-11268 and CCL-2; American Type Culture Collection (ATCC), Manassas, VA, USA) cells were cultured in Dulbecco’s modified Eagle’s medium (DMEM, 12800-017 Gibco, Thermo Fisher Scientific, Inc., Waltham, MA, USA) supplemented with 10% fetal bovine serum (FBS; cat. no. 12483-020L; Gibco; Thermo Fisher Scientific, Inc.) and 1% penicillin–streptomycin (cat.no. 15140122; Gibco, Thermo Fisher Scientific, Inc.). HCT116 cells were provided by Professor Albert J. Fornace (Georgetown University, Washington, DC, USA) and were grown in RPMI 1640 medium (cat. no. 1875-093; Gibco, Thermo Fisher Scientific, Inc.) supplemented with 10% FBS and 1% penicillin–streptomycin. All cells were maintained in a humidified incubator at 37 °C with 5% CO_2_. For transfections, the mixture of 10 mM polyethyleneimine reagent (cat. no. 23966, Polysciences, Inc., Warrington, PA, USA), 1–3 μg DNA, and 150 mM NaCl was used. The resulting transfection mixture was added to the cells, and the cells were incubated at 37 °C with 5% CO_2_.

### 4.2. Antibodies

The following antibodies were used for Western blotting, immunoprecipitation, and immunocytochemical staining: monoclonal anti-Bax (2D2) (1:1000 dilution; cat. no. sc-20067; Santa Cruz Biotechnology, Dallas, TX, USA), anti-Bax (6A7) (1:500 dilution; cat. no. sc-23959; Santa Cruz Biotechnology), anti-Bax (SPM336) (1:500 dilution; cat. no. sc-65532; Santa Cruz Biotechnology), anti-USP49 (1:1000 dilution; cat. no. 18066-1-AP; Proteintech, Rosemont, IL, USA), anti-Caspse-3 (1:1000 dilution for Caspase-3 and 1:500 dilution for Cleaved-Caspase-3; cat. no. 19677-1-AP; Proteintech), Caspase-9 (1:1000 dilution; cat.no. 10380-1-AP; Proteintech), anti-β-actin (1:4000 dilution; cat. no. sc-47778; Santa Cruz Biotechnology), anti-HA (1:1000 dilution; cat. no. 11583816001; Roche Diagnostics, Indianapolis, IN, USA), and anti-Flag (1:15,000 dilution; M185-3L, Sigma-Aldrich, St. Louis, MO, USA). For Western blotting, secondary antibodies used were mouse secondary antibody (1:30,000 dilution; cat. no. 62-6820, Invitrogen, Carlsbad, CA, USA) and rabbit secondary antibody (1:10,000 dilution; cat. no. sc-2357, Santa Cruze Biotechnology). To minimize the signal from IgG chains, Easy Blot antibody (1:1000 dilution, cat. no. GTX2219667-01; GeneTex, Irvine, CA, USA) was employed during immunoblotting. All antibodies were prepared in blocking buffers or antibody dilution solutions according to the manufacturer’s instructions and used as recommended.

### 4.3. Construction of Expression Vectors and Primers

To generate deletion mutants of *Bax* (1–219), *Bax* (220–334), and *Bax* (335–579), specific forward and reverse primers were designed and used for PCR amplification. The forward primers were 5′-GAA TTC GCA TGG ACG GGT-3′ for *Bax* (1–219), 5′-GAA TTC CGA TGG AGC TGC A-3′ for *Bax* (220–334), and 5′-GAA TTC GCA AAC TGG TGC TC-3′ for *Bax* (335–579). The corresponding reverse primers were 5′-CTC GAG CGG TTA CTG TCC AG-3′ for *Bax* (1–219), 5′-CTC GAG CCG CTG GCA AAG-3′ for *Bax* (220–334), and 5′-CTC GAG CGT CAG CCC ATC-3′ for *Bax* (335–579).

A point mutation of *USP49* (C262S) was introduced using site-directed mutagenesis. The forward primer 5′-GGC AAC ACC GCC TAC ATG AAC-3′ and reverse primer 5′-GTT CAT GTA GGC GGT GTT GCC-3′) were used to generate the mutant. Following PCR amplification and purification, the PCR product was treated with Dpn I enzyme (cat. no. R054S; Enzynomics Co., Ltd., Daejeon, Republic of Korea) and the construct was confirmed by sequencing.

To generate deletion mutants of USP49 (1–762), USP49 (763–1131), and USP49 (1132–2067), specific forward and reverse primers were designed and used for PCR amplification. The forward primers were 5′-GAA TTC GAT GGA TAG ATG C-3′ for USP49 (1–762), 5′-GAA TTC TCT GCG CAA CCT G-3′ for USP49 (763–1131), and 5′-TCT AGA ACC CTT CGC CAT GC-3′ for USP49 (1132–2067). The corresponding reverse primers were USP49 (1–762), 5′-CTC GAG CGA CAC TAG GGC-3′ for USP49 (763–1131), and 5′-TAC GTA TCA ACC CCT TTC C-3′ for USP49 (1132–2067).

To analyze the expression of USP49, we performed RT-PCR using specific forward and reverse primers. The forward primer 5′-AGG ACT ACG TGC TCA ATG ATA ACC-3′ and the reverse primer 5′-GCA GGA GCA GCC GTG CAC TCT-3′ were used to amplify USP49. Additionally, the forward primer 5′-ATC CCA TCA CCA TCT TCC-3′ and the revers primer 5′-CCA TCA CGC CAC AGT TTC-3′ were used to amplify the housekeeping gene *GAPDH*, serving as an internal control. cDNAs were transformed into the *Escherichia coli* DH5α (cat. no. RH617; RBC Bioscience Corp., New Taipei City, Taiwan) bacterial strain and the bacteria were grown for 16 h at 37 °C in Luria–Bertani broth (cat. no. MB-L4488; Kisan Bio Co., Ltd., Seoul, Republic of Korea).

*Yeast-two hybrid screening.* To identify protein–protein interactions, we employed the yeast two-hybrid screening method. The AH109 yeast strain was streaked on YPD (cat.no. 630464; Clontech Laboratories, Inc., Mountain View, CA, USA) agar plates and incubated at 30 °C for 3–4 days to obtain single colonies. The AH109 colony was then cultured in YPD media (cat.no. 30409; Clontech) and transformed using a lithium acetate (LiAc)-mediated method. In the LiAc transformation method, yeast cells with a density of 0.8–1.0 (OD600) were centrifuged at 2500 rpm for 2 min 30 s. The cells were resuspended in the LiAc solution and incubated at room temperature for 5 min, followed by centrifugation at 2500 rpm for 5 min. The cells were then resuspended with the LiAc solution. A mixture of plasmid DNA and carrier DNA was added to the suspended cells. Subsequently, 40% polyethylene glycol (PEG) in LiAc solution was added, and the mixture was incubated at 30 °C for 30 min. DMSO was added, and the cells were heat-shocked at 42 °C for 15 min. Finally, the cells were plated on appropriate medium, and transformants containing the plasmid were selected. For screening purposes, a sequential transformation method was employed. In the first transformation step, yeast cells were transformed with USP family members as bait and incubated on -Trp plates for 3–4 days. In second transformations, colonies were transformed with pGAD424-Bax and incubated on -Leu/-Trp minimal medium plates containing 4 mg/mL X-α-gal (cat. no. 630463; Clontech) to identify potential interacting partners. Positive colonies were further analyzed for protein–protein interactions.

### 4.4. Preparation of Cell Lysates, Western Blotting, and Immunoprecipitation

Cell lysates were prepared by washing cells with phosphate-buffered saline (PBS) and then lysing them in different buffers depending on the desired assay. For lysis, we used a RIPA lysis buffer composed of Tris-HCl [pH 7.5] 50 mM, NaCl 300 mM, EDTA 1 mM, glycerol 10%, and Triton X-100 1%. The lysates were incubated for 20 min on ice followed by centrifugation at 13,000 rpm for 20 min at 4 °C to remove insoluble materials. The resulting supernatant was collected for further analysis.

For Western blotting, 30 µg of protein from each sample was loaded onto an 8–12% SDS-PAGE and separated by electrophoresis. The proteins were then transferred onto polyvinylidene fluoride (PVDF) microporous membranes (cat. no. IPVH0010; MilliporeSigma, Burlington, MA, USA). The membranes were blocked in TBS-T (20 mM Tris-HCl [pH 7.5], 150 mM NaCl, 0.05% Tween 20) containing 5% skim milk for 20 min and incubated with primary antibodies at 4 °C overnight. After washing the membranes with TTBS, they were incubated with secondary antibodies in TTBS containing 2% skim milk (cat. no. 232100; Becton, Dickinson and Company, Franklin Lakes, NJ, USA) for 1 h. Following another round of washing, the membranes were visualized using an ECL reagent solution (Young In Frontier, Seoul, Republic of Korea). For immunoprecipitation, cell lysates were incubated with specific antibodies at 4 °C overnight. Protein A/G PLUS agarose beads (cat. no. sc-2003; Santa Cruz Biotechnology Inc.) were then added to the lysates and rotated for 2 h to facilitate antibody–protein complex formation. Following immunoprecipitation, samples were washed and boiled in 2X SDS sample buffer prior to Western blot analysis. This denaturing step was essential to disrupt non-covalent protein–protein interactions, ensuring that only covalently ubiquitinated Bax was detected.

### 4.5. GST Pull-Down Assay

BL21 (DE3) bacterial *Escherichia coli* strain (cat.no. C2527H; New England BioLabs, Ipswich, MA, USA) cells transformed with pGEX-4T-3 vector or pGEX-4T-3-Bax were cultured at 37 °C in Luria–Bertani broth. Once the cell density (OD_600_) reached 0.6, the expression of recombinant proteins was induced by adding 5 mM isopropyl β-D-1-thiogalactopyranoside (cat. no. V3955; Promega Corp., Madison, WI, USA) and incubating at 31 °C overnight. The cells were then lysed, and the resulting lysates containing the proteins of interest were mixed with Glutathione-Sepharose beads (cat. no. 17075605; Cytiva, Marlborough, MA, USA) and rotated. In parallel, HEK293T cells overexpressing Flag-USP49 were also lysed, and the cell extracts was mixed with GST and GST-Bax tagging with GST beads. The beads bound to the interacting proteins were washed to remove non-specifically bound molecules. The bound proteins were subsequently analyzed by Western blotting and probed with an anti-Flag antibody to detect the interaction between Flag-USP49 and the GST-tagged proteins. GST proteins were visualized using Coomassie Brilliant Blue (CBB) R250 and G250 solutions.

### 4.6. Immunocytochemical Staining and Confocal Microscopy

HCT116 cells were seeded on glass coverslips placed in a 12-well plate. The cells were fixed with 4% formaldehyde for 15 min and then blocked with a solution of PBS containing 2% normal goat serum and 1% Triton X-100 for 1 h at room temperature. Next, the cells were incubated with primary antibodies indicated above overnight at 4 °C. After washing with PBS, the cells were further incubated with Alexa-Fluor-488-cojugated goat anti-mouse (1:1000 dilution, Invitrogen) and Alexa-Fluor-568-conjugated goat anti-rabbit (1:1000 dilution, Invitrogen) for 1 h at room temperature. Finally, the samples were visualized using a confocal microscope (TCSSP5 II, Leica, Wetzlar, Germany).

### 4.7. Ubiquitination and Deubiquitination Assays

For the ubiquitination assay, HEK293T and HeLa cells were transfected with HA-ubiquitin, including HA-ubiquitin (K6), HA-ubiquitin (K11), HA-ubiquitin (K27), HA-ubiquitin (K29), HA-ubiquitin (K33), HA-ubiquitin (K48), and HA-ubiquitin (K63). After transfection, the cells were harvested, and cell lysates were prepared. Immunoprecipitation was performed using an anti-Bax antibody. For deubiquitination assay, cells were transfected with HA-ubiquitin and Flag-USP49 or *siUSP49* (5′–GCC GUA AUC AUC GAG AGA AGtt–3′) (GenePharma, Shanghai, China). Prior to cell harvest, the cells were treated with MG132 for 4 h. Immunoprecipitation was carried out using an anti-Bax antibody (1:1000 dilution; Santa Cruz Biotechnology) to precipitate proteins. The samples were then subjected to Western blotting analysis. The level of ubiquitination was determined using an anti-HA antibody to detect the presence of HA-tagged ubiquitin.

For the ubiquitination and deubiquitination assays, HA-Ub constructs were first transfected into a single large culture dish to ensure uniform transfection efficiency. On the following day, cells were trypsinized and evenly distributed into multiple plates. These plates were then transfected with either an empty vector (mock control) or Flag-USP49. This approach ensured that the initial expression levels of the HA-Ub constructs were consistent across all experimental groups prior to USP49 expression.

### 4.8. Cell Counting Kit-8 Assay

HeLa cells overexpressing Flag-*USP49* were treated with 1 μM STS and seeded into 96-well plates at a density of 6 × 10^3^ cells per well. The cells were incubated for 1–3 days to conduct the Cell Counting Kit-8 (CCK-8) assay (cat. no. CK04-01; DOJINDO Inc., Rockville, MD, USA). At 0, 24, 48, and 72 h, 100 μL of DMEM and 10 μL of CCK-8 solution were added to each well. The plates were then incubated for an additional 2 h to allow the reaction to proceed. Following this incubation period, absorbance values were measured at a wavelength of 450 nm.

### 4.9. Annexin V Staining and Cell Apoptosis Analysis

Annexin V staining and cell apoptosis analysis were performed with a fluorescence-activated cell sorting (FACS) and analyzed with CytoFLEX flow cytometer (Beckman Coulter, Inc., Brea, CA, USA) and CyExpert software (version 2.4.0.28; Beckman Coulter, Inc.). Apoptosis was determined by double staining with Annexin V-FITC and propidium iodide (PI) (Cat. no. #556547; BD BioScience, San Jose, CA, USA) according to the manufacturer’s instructions. Briefly, cells (1  ×  10^6^) were washed with cold PBS and then resuspended in a binding buffer (10 mM HEPES/NaOH (pH 7.4), 0.14 M NaCl, and 2.5 mM CaCl_2_). FITC Annexin V and PI solution were added to the cell suspension. After incubating at room temperature for 15 min in the dark, Annexin V-FITC and PI-staining were analyzed by flow cytometry. Cells stained with both Annexin V-FITC and PI were considered late apoptotic or necrotic.

## 5. Conclusions

In this study, we identify USP49 as a novel binding partner and regulator of the proapoptotic protein Bax. Through yeast two-hybrid screening, co-immunoprecipitation, GST pull-down assays, and immunocytochemical analyses, we demonstrate a robust and direct interaction between USP49 and Bax. Importantly, USP49 selectively modulates Bax polyubiquitination, with pronounced effects on K29-, K33-, and K63-linked ubiquitin chains, while exerting only minimal influence on K48-linked ubiquitination. Consistent with this ubiquitin linkage specificity, USP49 does not significantly alter Bax protein stability, indicating that its regulatory role is largely independent of proteasomal degradation.

Instead, our data reveal that USP49 increases *Bax* expression at the transcriptional level. This upregulation is maintained under multiple apoptotic stimuli, including UV irradiation and H_2_O_2_ treatment, suggesting that USP49-mediated transcriptional control of *Bax* occurs largely independently of stress-induced damage. Functionally, USP49 overexpression is associated with a consistent trend toward enhanced apoptosis, particularly under DNA damage conditions, although the magnitude of this effect varies depending on the apoptotic stimulus.

**Figure 6:**
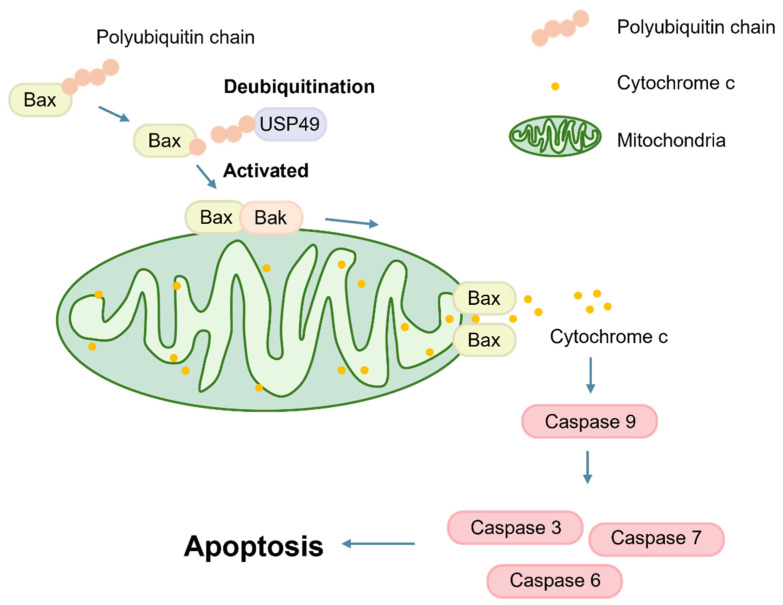
Proposed model of the USP49–Bax axis in apoptosis. USP49 interacts with Bax and modulates its ubiquitination status. This interaction correlates with Bax activation and mitochondrial translocation, where Bax, together with Bak, contributes to mitochondrial outer membrane permeabilization (MOMP). Subsequent mitochondrial signaling leads to the activation of initiator caspase-9 and downstream executioner caspases, including caspase-3, caspase-6, and caspase-7, ultimately resulting in apoptosis in solid cancer cells.

Collectively, these findings position USP49 as a non-canonical regulator of Bax that fine-tunes apoptotic signaling through non-degradative ubiquitin linkages and transcriptional upregulation rather than direct control of protein turnover (Figure 6). This study expands the current understanding of Bax regulation by DUBs and highlights the functional importance of atypical ubiquitin chain linkages in apoptosis. Further investigation of the USP49–Bax axis, including in vivo validation and identification of additional USP49 substrates, will be essential to clarify its broader role in stress responses and tumor biology, and to assess its potential as a therapeutic target in apoptosis-related diseases.

## Data Availability

The original contributions presented in this study are included in the article/Supplementary Materials. Further inquiries can be directed to the corresponding author.

## Supplementary Materials

The following supporting information can be downloaded at: https://www.mdpi.com/article/10.3390/ijms27094102/s1.

## Abbreviations

The following abbreviations are used in this manuscript:

AD	Activation domain
AKT	Protein kinase B
AML	Acute myeloid leukemia
ATCC	American type culture collection
BAG2	Bcl-2 associated athanogene 2
Bax	Bcl-2-associated X protein
Bcl-2	B-cell lymphoma 2
BD	Binding domain
BH	Bcl-2 homology
BTSA1	Bax trigger site activator 1
CBB	Coomassie brilliant blue
CCK-8	Cell counting kit-8
cDNA	Complementary DNA
CO_2_	Carbon dioxide
DMSO	Dimethyl sulfoxide
DNA	Deoxyribonucleic acid
Dpn I	Dpn I restriction enzyme
DSB	Double-strand break
DUB	Deubiquitinating enzyme
DMEM	Dulbecco’s modified Eagle’s medium
ECL	Enhanced chemiluminescence
EDTA	Ethylenediaminetetraacetic acid
*E. coli*	*Escherichia coli*
E1	Ubiquitin-activating enzyme
E2	Ubiquitin-conjugating enzyme
E3	Ubiquitin ligase
FACS	Fluorescence-activated cell sorting
FBS	Fetal bovine serum
FKBP51	FK506-binding protein 51
FITC	Fluorescein isothiocyanate
GAPDH	Glyceraldehyde-3-phosphate dehydrogenase
GPX4	Glutathione peroxidase 4
GST	Glutathione S-transferase
HA	Hemagglutinin (tag)
HEPES	4-(2-hydroxyethyl)-1-piperazineethanesulfonic acid
H	Hour(s)
H_2_O_2_	Hydrogen peroxide
IBRDC2	IBR domain-containing protein 2
IgG	Immunoglobulin G
IL-6	Interleukin 6
IP	Immunoprecipitation
IPTG	Isopropyl β-D-1-thiogalactopyranoside
JAMM	Jab1/MPN/Mov34 metalloenzyme
K (Ub)	Lysine residue (of ubiquitin)
K6/K11/K27/K29/K33/K48/K63Ubiquitin lysine linkage types	Ubiquitin lysine linkage types
Ku70	X-ray repair cross-complementing protein 6
LB	Luria–Bertani (broth)
LiAc	Lithium acetate
MG132	Proteasome inhibitor MG132
MJD	Machado–Josephin domain
MOM	Mitochondrial outer membrane
MOMP	Mitochondrial outer membrane permeabilization
mRNA	Messenger RNA
MCPIP	MCPIP
MYC (c-MYC)	Mitochondrial outer membrane permeabilization
OD600	Optical density at 600 nm
OTU	Ovarian tumor protease
PBS	Phosphate-buffered saline
PCR	Polymerase chain reaction
PEG	Polyethylene glycol
PEI	Polyethyleneimine
PI	Propidium iodide
PPPDE	Permutated papain fold peptidases of dsDNA viruses and eukaryote
PTM	Post-translational modification
PVDF	Polyvinylidene fluoride
qPCR	Quantitative polymerase chain reaction
RT-qPCR	Reverse transcription PCR
RPMI	Roswell Park Memorial Institute (medium)
RPM	Revolutions per minute
SDS-PAGE	Sodium dodecyl sulfate polyacrylamide gel electrophoresis
SHCBP1	SHC-binding and spindle-associated 1
SPM	Surgical pathology marker (antibody clone designation)
STAT3	Signal transducer and activator of transcription 3
STS	Staurosporine
SV40	Simian virus 40
TBS-T	Tris-buffered saline with Tween 20
TME	Tumor microenvironment
Tris-HCl	Tris(hydroxymethyl)aminomethane hydrochloride
Ub	Ubiquitin
UCH	Ubiquitin C-terminal hydrolase
UPS	Ubiquitin–proteasome system
USP	Ubiquitin-specific protease
USP49	Ubiquitin-specific protease 49
UV	Ultraviolet
WB	Western blotting
WT	Wild-type
X-α-gal	5-bromo-4-chloro-3-indolyl-α-D-galactopyranoside
YAP1	Yes-associated protein 1
YPD	Yeast extract–peptone–dextrose
ZUFSP/ZUP1	ZUFSP (zinc finger with UFM1-specific peptidase domain)/ZUP1 subfamily

## References

[1] Czabotar P.E., Garcia-Saez A.J. (2023). Mechanisms of BCL-2 family proteins in mitochondrial apoptosis. Nat. Rev. Mol. Cell Biol..

[2] Dadsena S., Cuevas Arenas R., Vieira G., Brodesser S., Melo M.N., Garcia-Saez A.J. (2024). Lipid unsaturation promotes BAX and BAK pore activity during apoptosis. Nat. Commun..

[3] Khadrawy S.M., Altoom N.G., Alotaibi A.G., Othman S.I. (2024). Hepatoprotective potential of taxifolin in type 2 diabetic rats: Modulation of oxidative stress and Bcl2/Bax/Caspase-3 signaling pathway. Mol. Biol. Rep..

[4] Westphal D., Dewson G., Czabotar P.E., Kluck R.M. (2011). Molecular biology of Bax and Bak activation and action. Biochim. Biophys. Acta.

[5] Moldoveanu T., Czabotar P.E. (2020). BAX, BAK, and BOK: A coming of age for the BCL-2 family effector proteins. Cold Spring Harb. Perspect. Biol..

[6] Tchernev G., Orfanos C.E. (2007). Downregulation of cell cycle modulators p21, p27, p53, Rb and proapoptotic Bcl-2-related proteins Bax and Bak in cutaneous melanoma is associated with worse patient prognosis: Preliminary findings. J. Cutan. Pathol..

[7] Guner D., Sturm I., Hemmati P., Hermann S., Hauptmann S., Wurm R., Budach V., Dorken B., Lorenz M., Daniel P.T. (2003). Multigene analysis of Rb pathway and apoptosis control in esophageal squamous cell carcinoma identifies patients with good prognosis. Int. J. Cancer.

[8] Li B., Dou Q.P. (2000). Bax degradation by the ubiquitin/proteasome-dependent pathway: Involvement in tumor survival and progression. Proc. Natl. Acad. Sci. USA.

[9] Reyna D.E., Garner T.P., Lopez A., Kopp F., Choudhary G.S., Sridharan A., Narayanagari S.R., Mitchell K., Dong B., Bartholdy B.A. (2017). Direct activation of BAX by BTSA1 overcomes apoptosis resistance in acute myeloid leukemia. Cancer Cell.

[10] Rape M. (2018). Ubiquitylation at the crossroads of development and disease. Nat. Rev. Mol. Cell Biol..

[11] Clague M.J., Urbe S., Komander D. (2019). Breaking the chains: Deubiquitylating enzyme specificity begets function. Nat. Rev. Mol. Cell Biol..

[12] Swatek K.N., Komander D. (2016). Ubiquitin modifications. Cell Res..

[13] Panier S., Durocher D. (2009). Regulatory ubiquitylation in response to DNA double-strand breaks. DNA Repair.

[14] Amerik A.Y., Hochstrasser M. (2004). Mechanism and function of deubiquitinating enzymes. Biochim. Biophys. Acta.

[15] Nijman S.M., Luna-Vargas M.P., Velds A., Brummelkamp T.R., Dirac A.M., Sixma T.K., Bernards R. (2005). A genomic and functional inventory of deubiquitinating enzymes. Cell.

[16] Choi H.S., Baek K.H. (2022). Pro-apoptotic and anti-apoptotic regulation mediated by deubiquitinating enzymes. Cell Mol. Life Sci..

[17] Abdul Rehman S.A., Kristariyanto Y.A., Choi S.Y., Nkosi P.J., Weidlich S., Labib K., Hofmann K., Kulathu Y. (2016). MINDY-1 is a member of an rvolutionarily conserved and structurally distinct new family of deubiquitinating enzymes. Mol. Cell.

[18] Wilkinson K.D. (1987). Protein ubiquitination: A regulatory post-translational modification. Anticancer. Drug Des..

[19] Benard G., Neutzner A., Peng G., Wang C., Livak F., Youle R.J., Karbowski M. (2010). IBRDC2, an IBR-type E3 ubiquitin ligase, is a regulatory factor for Bax and apoptosis activation. EMBO J..

[20] Johnson B.N., Berger A.K., Cortese G.P., Lavoie M.J. (2012). The ubiquitin E3 ligase parkin regulates the proapoptotic function of Bax. Proc. Natl. Acad. Sci. USA.

[21] Shen J., Yang H., Qiao X., Chen Y., Zheng L., Lin J., Lang J., Yu Q., Wang Z. (2023). The E3 ubiquitin ligase TRIM17 promotes gastric cancer survival and progression via controlling BAX stability and antagonizing apoptosis. Cell Death Differ..

[22] Wang S.A., Wang Y.C., Chuang Y.P., Huang Y.H., Su W.C., Chang W.C., Hung J.J. (2017). EGF-mediated inhibition of ubiquitin-specific peptidase 24 expression has a crucial role in tumorigenesis. Oncogene.

[23] Matsuyama S., Palmer J., Bates A., Poventud-Fuentes I., Wong K., Ngo J., Matsuyama M. (2016). Bax-induced apoptosis shortens the life span of DNA repair defect Ku70-knockout mice by inducing emphysema. Exp. Biol. Med..

[24] Cohen H.Y., Lavu S., Bitterman K.J., Hekking B., Imahiyerobo T.A., Miller C., Frye R., Ploegh H., Kessler B.M., Sinclair D.A. (2004). Acetylation of the C terminus of Ku70 by CBP and PCAF controls Bax-mediated apoptosis. Mol. Cell.

[25] Wang A., Ning Z., Lu C., Gao W., Liang J., Yan Q., Tan G., Liu J. (2017). USP22 Induces Cisplatin Resistance in Lung Adenocarcinoma by Regulating γH2AX-Mediated DNA Damage Repair and Ku70/Bax-Mediated Apoptosis. Front. Pharmacol..

[26] Choi H.S., Lim E.S., Baek K.H. (2022). Deubiquitinating enzyme USP12 regulates the pro-apoptosis protein Bax. Int. J. Mol. Sci..

[27] Kwon S.K., Lee D.H., Kim S.Y., Park J.H., Choi J., Baek K.H. (2017). Ubiquitin-specific protease 21 regulating the K48-linked polyubiquitination of NANOG. Biochem. Biophys. Res. Commun..

[28] Herhaus L., Dikic I. (2015). Expanding the ubiquitin code through post-translational modification. EMBO Rep..

[29] Hershko A., Ciechanover A. (1998). The ubiquitin system. Annu. Rev. Biochem..

[30] Hanpude P., Bhattacharya S., Dey A.K., Maiti T.K. (2015). Deubiquitinating enzymes in cellular signaling and disease regulation. IUBMB Life.

[31] Baig S., Seevasant I., Mohamad J., Mukheem A., Huri H.Z., Kamarul T. (2016). Potential of apoptotic pathway-targeted cancer therapeutic research: Where do we stand?. Cell Death Dis..

[32] Plati J., Bucur O., Khosravi-Far R. (2011). Apoptotic cell signaling in cancer progression and therapy. Integr. Biol..

[33] Amirghofran Z., AMonabati Gholijani N. (2005). Apoptosis in prostate cancer: Bax correlation with stage. Int. J. Urol..

[34] Friess H., Lu Z., Graber H.U., Zimmermann A., Adler G., Korc M., Schmid R.M., Buchler M.W. (1998). bax, but not bcl-2, influences the prognosis of human pancreatic cancer. Gut.

[35] Pluta P., Smolewski P., Pluta A., Cebula-Obrzut B., Wierzbowska A., Nejc D., Robak T., Kordek R., Gottwald L., Piekarski J. (2011). Significance of Bax expression in breast cancer patients. Pol. Przegl Chir..

[36] Agrawal S.G., Liu F.T., Wiseman C., Shirali S., Liu H., Lillington D., Du M.Q., Syndercombe-Court D., Newland A.C., Gribben J.G. (2008). Increased proteasomal degradation of Bax is a common feature of poor prognosis chronic lymphocytic leukemia. Blood.

[37] Zhang Z., Jones A., Joo H.Y., Zhou D., Cao Y., Chen S., Erdjument-Bromage H., Renfrow M., He H., Tempst P. (2013). USP49 deubiquitinates histone H2B and regulates cotranscriptional pre-mRNA splicing. Genes Dev..

[38] Luo K., Li Y., Yin Y., Li L., Wu C., Chen Y., Nowsheen S., Hu Q., Zhang L., Lou Z. (2017). USP49 negatively regulates tumorigenesis and chemoresistance through FKBP51-AKT signaling. EMBO J..

[39] Shi L., Shen X., Shen Y. (2022). USP49-Mediated Histone H2B Deubiquitination Regulates HCT116 Cell Proliferation through MDM2-p53 Axis. Mol. Cell Biol..

[40] Tu R., Kang W., Yang X., Zhang Q., Xie X., Liu W., Zhang J., Zhang X.D., Wang H., Du R.L. (2018). USP49 participates in the DNA damage response by forming a positive feedback loop with p53. Cell Death Dis..

[41] Tu R., Kang W., Kang Y., Chen Z., Zhang P., Xiong X., Ma J., Du R.L., Zhang C. (2022). c-MYC-USP49-BAG2 axis promotes proliferation SSand chemoresistance of colorectal cancer cells in vitro. Biochem. Biophys. Res. Commun..

[42] Xu W., Shan J., Wang J., Zhou Y., Ouyang Y., Chen Y., Zhou Q. (2026). USP49 regulates lipid metabolism in hepatocellular carcinoma by stabilizing RACK1 to promote tumor proliferation and migration. Biochim. Biophys. Acta Mol. Cell Res..

[43] Gardai S.J., Hildeman D.A., Frankel S.K., Whitlock B.B., Frasch S.C., Borregaard N., Marrack P., Bratton D.L., Henson P.M. (2004). Phosphorylation of Bax Ser184 by Akt regulates its activity and apoptosis in neutrophils. J. Biol. Chem..

[44] Tsuruta F., Masuyama N., Gotoh Y. (2002). The phosphatidylinositol 3-kinase (PI3K)-Akt pathway suppresses Bax translocation to mitochondria. J. Biol. Chem..

[45] Yamaguchi H., Wang H.G. (2001). The protein kinase PKB/Akt regulates cell survival and apoptosis by inhibiting Bax conformational change. Oncogene.

[46] Matsui M., Kajita S., Tsuchiya Y., Torii W., Tamekuni S., Nishi R. (2022). USP49 is a novel deubiquitylating enzyme for γ H2AX in DNA double-strand break repair. Gene.

[47] Liu Z., Li J., Ding Y., Ma M., Chen J., Lei W., Li L., Yao Y., Yu X., Zhong M. (2022). USP49 mediates tumor progression and poor prognosis through a YAP1-dependent feedback loop in gastric cancer. Oncogene.

[48] Ding Y., Liu Z., Dai X., Ruan R., Zhong H., Wu Z., Yao Y., Chen J., Deng J., Xiong J. (2024). USP49 promotes adenocarcinoma of the esophagogastric junction malignant progression via activating SHCBP1-beta-catenin-GPX4 axis. Carcinogenesis.

[49] Wu Q., Lv J., Li X. (2025). Membrane palmitoylated protein MPP1 inhibits immune escape by regulating the USP12/CCL5 axis in urothelial carcinoma. Int. Immunopharmacol..

[50] Ye L., Zhang Q., Liuyu T., Xu Z., Zhang M.X., Luo M.H., Zeng W.B., Zhu Q., Lin D., Zhong B. (2019). USP49 negatively regulates cellular antiviral responses via deconjugating K63-linked ubiquitination of MITA. PLoS Pathog..

[51] Chargui A. (2024). Lysine-63-linked polyubiquitination: A principal target of cadmium carcinogenesis. Toxicol. Res..

[52] Wickliffe K.E., Williamson A., Meyer H.J., Kelly A., Rape M. (2011). K11-linked ubiquitin chains as novel regulators of cell division. Trends Cell Biol..

[53] Tracz M., Bialek W. (2021). Beyond K48 and K63: Non-canonical protein ubiquitination. Cell Mol. Biol. Lett..

