# Supplementary material for: Ubiquitin-Specific Protease 49 Interacts with Bax to Modulate Apoptosis
Source: Int J Mol Sci. 2026 May 3;27(9):4102. doi: 10.3390/ijms27094102 (PMC13164060; doi:10.3390/ijms27094102)
Supplement: Supplementary file 1 [file ijms-27-04102-s001.zip › ijms-4176976-supplementary.pdf]

Supplementary data

S1

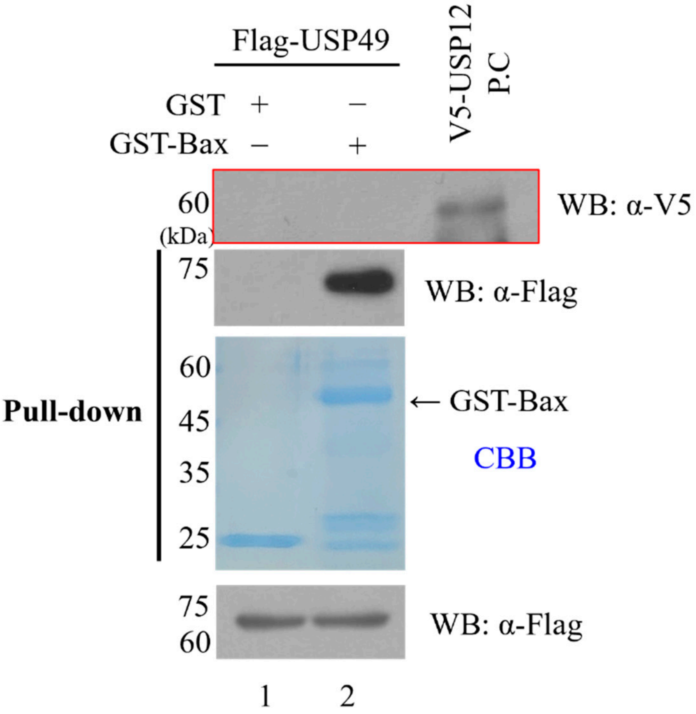

Supplementary Figure S1. Direct interaction between USP49 and Bax *in vitro*. GST pull-down assay was performed to examine the direct interaction between USP49 and Bax. Purified recombinant GST/GST-Bax proteins were immobilized on glutathione-agarose beads and incubated with purified Bax or USP12. After extensive washing, the bound proteins were eluted, resolved by SDS-PAGE, and analyzed by immunoblotting (IB) with an anti-Flag or V5 antibody. The input lane represents a portion of the total recombinant protein used in the binding reaction. Equal loading of the GST and GST-fused proteins was confirmed by Coomassie brilliant blue (CBB) staining.

S2

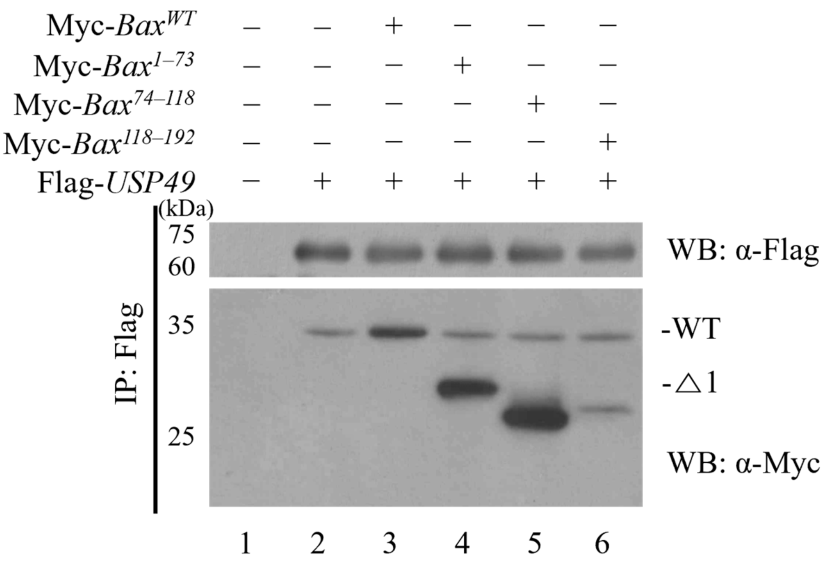

Supplementary Figure S2. Co-immunoprecipitation of USP49 and Bax truncation mutants. HeLa cells were transfected with Flag-USP49 alone or co-transfected with Myc-Bax WT or its truncation mutants. Cell lysates were immunoprecipitated (IP) with an anti-Flag antibody and immunoblotted (IB) with an anti-Myc antibody. The full blot is shown, including a negative control lane (Flag-USP49 transfected alone without Myc-Bax) to demonstrate that the faint band observed at approximately 33 kDa is a non-specific background signal. The specific interaction signal for WT Myc-Bax is significantly more intense than this baseline noise.

S3

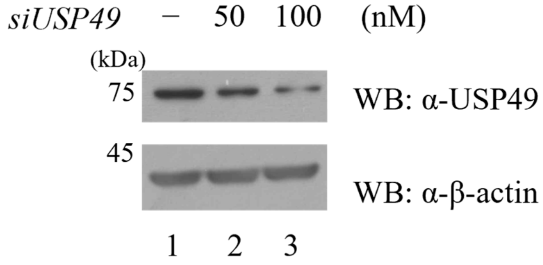

Supplementary Figure S3. Effect of si*USP49* on USP49 knockdown. HeLa cells were co-transfected with Myc-Bax and HA-Ub, along with negative control siRNA (si-NC) or increasing concentrations of USP49 siRNA (50 and 100 nM). Bax ubiquitination was analyzed by immunoprecipitation (IP) with an anti-Myc antibody followed by immunoblotting (IB) with an anti-HA antibody under denaturing conditions. The knockdown efficiency of USP49 was confirmed by Western blotting of whole cell lysates (WCL). β-actin was used as a loading control. The results show that the polyubiquitination of Bax increases in a dose-dependent manner as USP49 expression is progressively reduced.
